# Prmt7 promotes myoblast differentiation via methylation of p38MAPK on arginine residue 70

**DOI:** 10.1038/s41418-019-0373-y

**Published:** 2019-06-26

**Authors:** Hyeon-Ju Jeong, Sang-Jin Lee, Hye-Jin Lee, Hye-Been Kim, Tuan Anh Vuong, Hana Cho, Gyu-Un Bae, Jong-Sun Kang

**Affiliations:** 1 Department of Molecular Cell Biology, Sungkyunkwan University School of Medicine, Single Cell Network Research Center, Sungkyunkwan University, Suwon, 16419 Republic of Korea; 2 Drug Information Research Institute, College of Pharmacy, Sookmyung Women’s University, Seoul, 04310 Republic of Korea; 3 Department of Physiology, Sungkyunkwan University School of Medicine, Single Cell Network Research Center, Sungkyunkwan University, Suwon, 16419 Republic of Korea

**Keywords:** Proteins, Epigenetics

## Abstract

MyoD functions as a master regulator to induce muscle-specific gene expression and myogenic differentiation. Here, we demonstrate a positive role of Protein arginine methyltransferase 7 (Prmt7) in MyoD-mediated myoblast differentiation through p38MAPK activation. Prmt7 depletion in primary or C2C12 myoblasts impairs cell cycle withdrawal and myogenic differentiation. Furthermore, Prmt7 depletion decreases the MyoD-reporter activities and the MyoD-mediated myogenic conversion of fibroblasts. Together with MyoD, Prmt7 is recruited to the *Myogenin* promoter region and Prmt7 depletion attenuates the recruitment of MyoD and its coactivators. The mechanistic study reveals that Prmt7 methylates p38MAPKα at the arginine residue 70, thereby promoting its activation which in turn enhances MyoD activities. The arginine residue 70 to alanine mutation in p38MAPKα impedes MyoD/E47 heterodimerization and the recruitment of Prmt7, MyoD and Baf60c to the *Myogenin* promoter resulting in blunted Myogenin expression. In conclusion, Prmt7 promotes MyoD-mediated myoblast differentiation through methylation of p38MAPKα at arginine residue 70.

## Introduction

Skeletal muscle regeneration proceeds through multiple steps, including activation of quiescent satellite cells, amplification of activated progenitors, and terminal differentiation and fusion of myoblasts into preexisting myofibers [1, 2]. Myoblast differentiation is regulated by transcription factors belonging to myogenic basic helix-loop-helix (bHLH) family (MyoD, Myf5, Myogenin and MRF4) and Myocyte enhancer factor 2 (Mef2) which cooperate to induce muscle-specific gene expression and terminal differentiation of myoblasts [3, 4]. MyoD family functions as master regulators to induce muscle-specific gene expression and myogenic differentiation in non-muscle cells, like fibroblasts [5, 6]. One of the key events for MyoD activation is the heterodimerization with its partner E proteins (E12 and E47) that can bind to the consensus DNA sequence called E-box (CANNTG) found in regulatory regions of many muscle-specific genes [7–10]. Among signaling pathways, p38MAPK plays a key role in myogenesis through activation of MyoD-mediated gene expression via phosphorylation of several transcriptional regulators including E proteins and chromatin-modifying enzyme SWI/SNF subunit Baf60c [11–14].

Protein arginine methyltransferases (Prmts) catalyze symmetric or asymmetric dimethylation of arginine residues on both histone and non-histone substrates to modulate signaling pathways and gene expression involved in diverse biological processes, including myoblast differentiation [15–17]. Depending on the methylation property, Prmts can be classified as the type I catalyzing asymmetric arginine dimethylation (Prmt1, Prmt2, Prmt3, Prmt4, Prmt6, and Prmt8) or the type II subfamily generating symmetric dimethyl-arginine residues (Prmt5, Prmt7 and Prmt9) [15, 16]. Prmt4 and Prmt5 have been implicated in promotion of myoblast differentiation through interaction with Mef2c or MyoD, respectively [18, 19]. In myogenesis, MyoD-mediated transcription requires the coordinated recruitment of histone modifying enzymes and ATP-dependent chromatin remodeling proteins, such as Brg1 and Baf subunit of SWI/SNF complexes [20]. Consistently, Prmt5 is recruited to the *Myogenin* promoter region with Brg1 and induces symmetric dimethylation of histone H3R8 in MyoD-induced myoblast differentiation. Prmt5 depletion leads to the abrogation of Brg1 and MyoD recruitment, accompanied by decreased histone H3R8 dimethylation [19]. Like Prmt5, Prmt7 generates symmetric dimethyl-arginine residues of histone or nonhistone substrates [21, 22]. Prmt7 has been implicated in diverse biological processes, including repression of DNA damage response, repression of E-cadherin inducing epithelial-to-mesenchymal transition in breast cancer cell lines [1, 23]. In the DNA damage response, Prmt7 interacts with Brg1 and Baf subunits of SWI/SNF chromatin remodeling proteins to suppress DNA repair gene expression through symmetric dimethylation of histone H2AR3 and histone H4R3 at the target DNA repair genes [1]. In addition, both Prmt7 and Prmt5 are recently found in euchromatic regions and mediate symmetric methylation of histone H3R2, thereby facilitating the recruitment of transcription regulators in cell differentiation [21]. Both Prmt5 and Prmt7 are expressed in muscles and during myoblast differentiation [19] and share common binding partners, such as Brg1 and Baf60 [1], which also play critical roles in MyoD-mediated gene expression during myoblast differentiation.

Recent studies with satellite cell-specific deletion mouse models for Prmt1, Prmt4, Prmt5, and Prmt7 have underlined the importance of arginine methylation in muscle regeneration. Prmt4 regulates Myf5 induction through methylation of Pax7 during asymmetric division of satellite cells [24]. Prmt5 is involved in muscle stem cell proliferation by silencing of a cell cycle inhibitor p21 [25]. Prmt1-deficient satellite cells exhibit enhanced proliferation with defective terminal differentiation [26]. A recent study has reported that Prmt7 deficiency impairs muscle differentiation and regeneration. Prmt7-deficient satellite cells enter into cellular senescence upon activation due to diminished expression of DNA methyltransferase 3b (DNMT3b) and a consequential increase in p21 [27]. However, the detailed mechanisms and nonhistone substrates by which Prmt7 regulates myogenic differentiation is currently unknown. In this study, we examine the role and mechanism of Prmt7 in myoblast differentiation. We demonstrate a promyogenic role of Prmt7 that augments MyoD-mediated myogenic differentiation through p38MAPK activation. The arginine residue 70 of p38MAPK is the critical target of Prmt7 in MyoD activation and myoblast differentiation.

## Results

### Prmt7 deficiency causes impaired myogenic differentiation

**Fig. 1:**
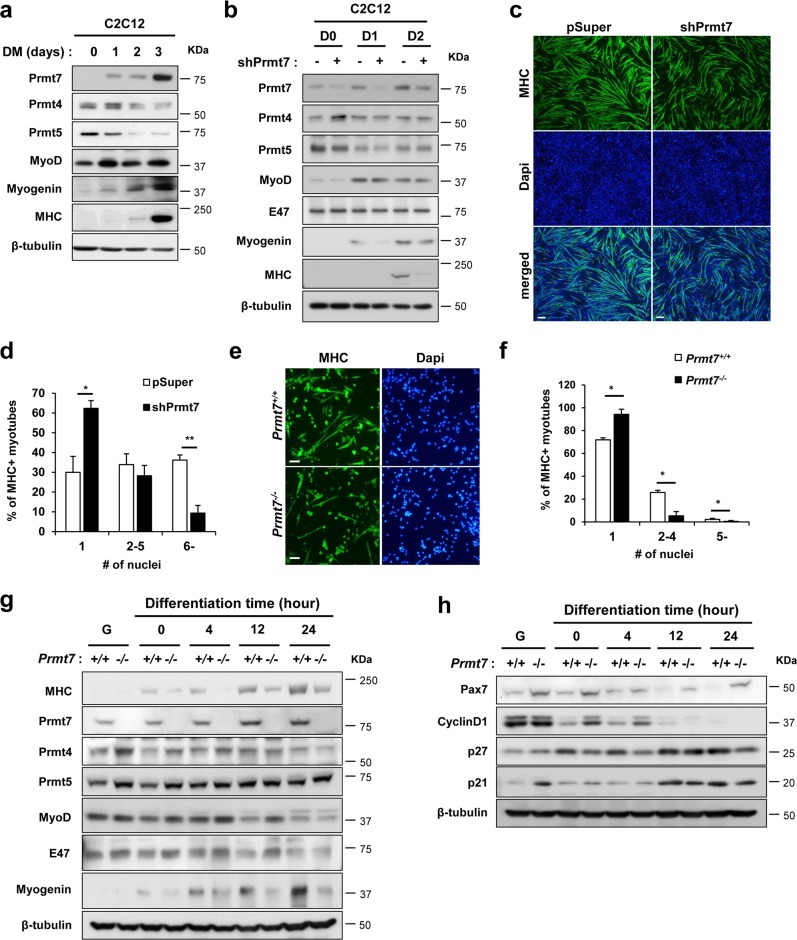
Prmt7 Deficiency Causes Impaired Myoblasts Differentiation. **a** Immunoblot analysis of C2C12 cells from D0 to D3 was performed for the expression of Prmt7, Prmt4, Prmt5, or myogenic genes and β-tubulin serves as loading control. **b** C2C12/pSuper and C2C12/shPrmt7 cells were induced to differentiate for indicated differentiation days followed by immunoblot analysis for the expression of Prmt7, Prmt4, Prmt5, muscle markers and E47. β-Tubulin serves as loading control. The experiment was repeated three times with similar results. **c** C2C12/pSuper and C2C12/shPrmt7 cells were induced to differentiate for 3 days and immunostained with anti-MHC antibodies, followed by Dapi staining to visualize nuclei. Size bar, 100μm. **d** The quantification of myotube formation shown in the panel **c**. Values represent means of random five field ± SD. The experiment was repeated three times with similar results. Significant difference from control, *P < 0.05, **P < 0.01. **e** *Prmt7*^*+/+*^ and *Prmt7*^*−/−*^ myoblasts were induced to differentiate for 24 h and immunostained for MHC, followed by Dapi staining to visualize nuclei. Size bar, 100 μm. **f** The quantification of MHC-positive cells and number of nuclei per myotube. Values represent means of triplicates ± SD. Significant difference from control, *P < 0.05. **g**, **h** *Prmt7*^*+/+*^ and *Prmt7*^*−/−*^ myoblasts were differentiate for 24 h and lysates were subjected to immunoblotting. The experiment was repeated twice with similar results

To determine the molecular mechanism of Prmt7 in myogenic differentiation, we have employed C2C12 and primary myoblasts isolated from wildtype or Prmt7-deficient mice. C2C12 cells were induced to differentiate and analyzed for the expression of Prmt7, Prmt4, Prmt5 and myogenic markers, MyoD, Myogenin and myosin heavy chain (MHC). Similarly to the expression pattern of Myogenin and MHC, Prmt7 was enhanced at differentiation day 1 (D1) and further increased at D3, while Prmt4 and Prmt5 levels were gradually reduced during differentiation (Fig. 1a and S1a). C2C12 cells were stably transfected with control pSuper or Prmt7 shRNA (shPrmt7) vectors and their differentiation was assessed by immunoblotting and MHC immunostaining (Fig. 1b, c and S1b). Prmt7 induction during differentiation was blunted by shPrmt7 expression. Prmt7 depletion reduced the expression of Myogenin and MHC, relative to control. Prmt5, MyoD and E47 levels did not differ between control and Prmt7-depleted myoblasts, while Prmt4 was increased in Prmt7-depleted myoblasts at D0 (Fig. 1b and S1b). Prmt7-depleted cells at D3 formed smaller MHC-positive myotubes with fewer nuclei, compared to control cells (Fig. 1c, d).

To further confirm, we have isolated primary myoblasts from hindlimbs of *Prmt7*^*+/+*^ or *Prmt7*^*−/−*^ mice and induced to differentiate for 2 days, followed by MHC immunostaining and immunoblotting for myogenic markers. Prmt7-deficient myoblasts formed more mononucleated myocytes and less myotubes containing multinuclei, compared to wildtype cells (Fig. 1e, f). Consistently, MHC and Myogenin levels were greatly decreased in Prmt7-deficient myoblasts upon differentiation without affecting MyoD and E47 levels, while Prmt4 and Prmt5 levels were increased in early differentiation (Fig. 1g and S1c). Myogenin expression was significantly reduced in differentiating *Prmt7*^*−/−*^ myoblasts, while MyoD levels were unaltered relative to wildtype myoblasts (Figure S2). Upon differentiation induction, *Prmt7*^*+/+*^ myoblasts displayed reduced Pax7 and Cyclin D1 expression (Fig. 1h and S1d). However, *Prmt7*^*−/−*^ myoblasts maintained high levels of Pax7 in differentiation condition and Cyclin D1 expression diminished slowly in *Prmt7*^*−/−*^ myoblasts, relative to *Prmt7*^*+/+*^ myoblasts. Interestingly, a cell cycle inhibitor p27 levels were gradually increased in *Prmt7*^*+/+*^ myoblasts while no obvious increase of p27 until 12 h post differentiation induction was observed in *Prmt7*^*−/−*^ myoblasts. A previous study has reported that Prmt7 deficiency impairs the proliferative capacity associated with cellular senescence through p21 upregulation [27]. Consistently, *Prmt7*^*−/−*^ myoblasts in growing condition with freshly added basic fibroblast growth factor had greatly elevated p21 levels, relative to *Prmt7*^*+/+*^ myoblasts. However, p21 initially decreased upon differentiation and after 12 h of differentiation induction it was starkly enhanced in both wildtype and *Prmt7*^*−/−*^ myoblasts (Fig. 1h and S1d). Thus, p21 induction specifically related to differentiation is not greatly altered in Prmt7-deficient myoblasts. In addition, Prmt7 depletion by shRNA-expressing lentiviruses resulted in increased number of Pax7-positive cells on single myofibers, compared to control virus-infected myofibers (Figure S3a–S3c). Furthermore, the cell cycle profiling of wildtype and *Prmt7*^*−/−*^ myoblasts at 12 h post differentiation induction revealed that Prmt7-deficient cells had a reduction in G1 phase with more cells in the S and M phase, compared with wildtype cells (Figure S3d). These results suggest that Prmt7 deficiency impairs myoblast differentiation accompanied by perturbed cell cycle arrest.

### Mice lacking Prmt7 exhibit delayed muscle regeneration

**Fig. 2:**
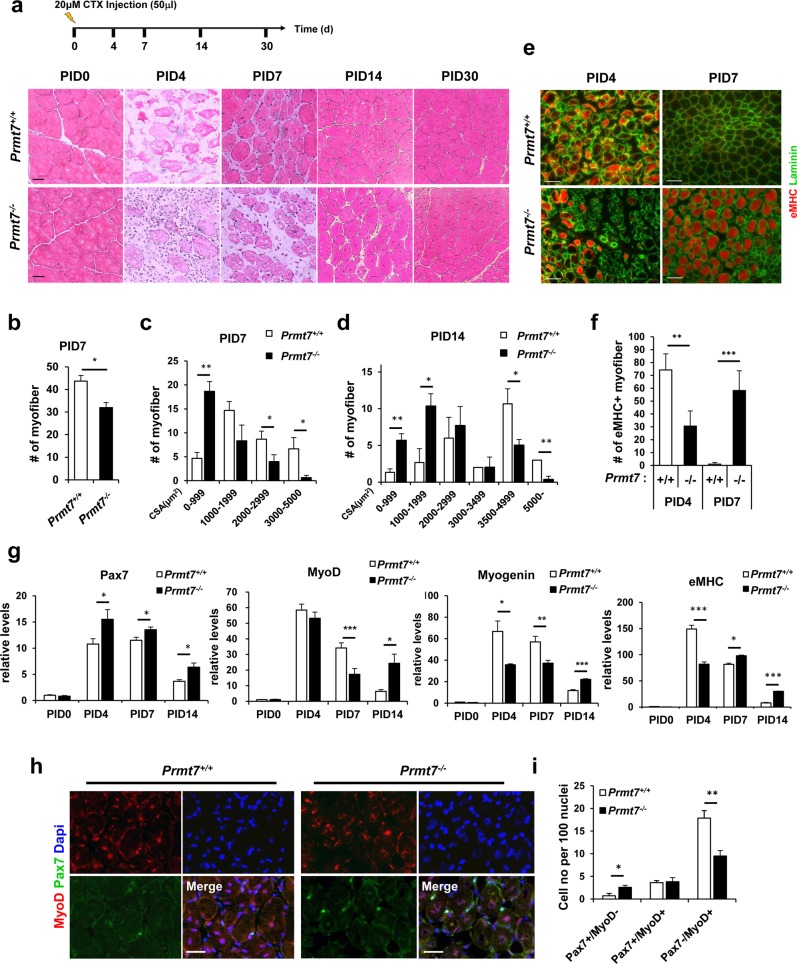
Mice Lacking Prmt7 Exhibit Delayed Muscle Regeneration. **a** The scheme of cardiotoxin injury and representative images of hematoxylin and eosin stained sections of TA muscles from *Prmt7*^*+/+*^ and *Prmt7*^*−/−*^ mice harvested at 0, 4, 7, 14 and 30 days post cardiotoxin injury (PID). Scale bar, 20μm. **b** Quantification of the number of eosin-positive myofibers from *Prmt7*^*+/+*^ and *Prmt7*^*−/−*^ TA muscles at 7 days after injury. **c**, **d** Quantification of the cross-sectional area of *Prmt7*^*+/+*^ and *Prmt7*^*−/−*^ TA muscles at 7 and 14 days following injury. Values are mean ± SEM (n = 3 mice per group). *P < 0.05, **P *<* 0.01. **e** Immunostaining of TA muscles from *Prmt7*^*+/+*^ and *Prmt7*^*−/−*^ mice with anti- eMHC and anti-laminin antibodies at 4 and 7 days post injury. **f** Quantification of the number of eMHC-positive myofibers. Values are means ± SD. **P < 0.01, ***P < 0.001 (*n* = 3). **g** Quantitative RT-PCR analysis for various myogenic markers in *Prmt7*^*+/+*^ and *Prmt7*^*−/−*^ TA muscles at indicated time points (post injury days 0, 4, 7 and 14) after CTX injury. Markers analyzed are Pax7, MyoD, Myogenin and eMHC. Values are means ± SEM. *P < 0.05, **P < 0.01, ***P < 0.001 (*n* = 3). **h** Immunostaining of TA muscles from *Prmt7*^*+/+*^ and *Prmt7*^*−/−*^ mice at 7 days after CTX injury with anti-Pax7 and anti-MyoD antibodies. **i** Quantification of the number of cells undergoing self-renewal (Pax7^+^/MyoD^-^), activation (Pax7^+^/MyoD^+^) and differentiation (Pax7^-^/MyoD^+^). Values are means ± SD. *P < 0.05, **P < 0.01 (*n* = 3)

To further examine the role of Prmt7 in muscle regeneration, tibialis anterior (TA) muscles of four-month-old wildtype and Prmt7-deficient mice were injured by cardiotoxin injection, followed by sampling muscles at the indicated time points. Muscle structure was examined by hematoxylin and eosin staining (Fig. 2a). Wildtype muscles exhibited necrotic myofibers at post injury 4 days (PID4), which recovered with newly regenerating myofibers with centrally localized myonuclei at PID7. At PID14, the fiber size returned to that of PID0. In contrast, Prmt7-deficient muscles showed delayed regeneration. At PID14, small myofibers and interstitial spaces were observed and fibers with centrally localized myonuclei were still present at PID30 (Fig. 2a). The quantification of the number and cross-sectional area of myofibers in muscles at PID7 and PID14 revealed that Prmt7-deficient muscles had fewer and smaller newly formed myofibers, compared to those of wildtype (Fig. 2b–d). Additionally, newly regenerating myofibers in wildtype muscles at PID4 were positive for an immature myofiber marker embryonic myosin heavy chain (eMHC) (Fig. 2e, f). Consistently, wildtype myofibers at PID7 had greatly reduced eMHC-positive fibers. In contrast, Prmt7-deificent muscles at PID4 had few eMHC-positive myofibers but the majority of myofibers at PID7 was still positive for eMHC (Fig. 2e, f). In consistent with the previous report [27], these data further support for a critical role of Prmt7 in muscle regeneration.

TA muscles were subjected to quantitative RT-PCR analysis for the expression of Pax7, MyoD, Myogenin, and eMHC (Fig. 2g). At PID4, Pax7 was dramatically increased and stayed high until PID7 which then decreased at PID14 in both wildtype and *Prmt7*^*−/−*^ muscles. However, Pax7 was significantly higher in *Prmt7*^*−/−*^ regenerating muscles at all examined time points. At PID4, the differentiation markers, MyoD, Myogenin and eMHC were greatly induced and slowly reduced thereafter in wildtype muscles (Fig. 2g). Prmt7-deficient muscles showed a similar expression pattern for MyoD at PID4, while Myogenin was significantly lower, compared to wildtype. Considering that MyoD activity is critical for Myogenin induction, these data suggest that Prmt7 deficiency leads to decreased MyoD activity. At PID7, Prmt7-deficient muscles had decreased MyoD and Myogenin levels which were significantly increased at PID14, compared to the wildtype. In consistent with the immunostaining data, eMHC expression was significantly decreased in Prmt7-deficient muscles at PID4, however it stayed high until PID14. The immunostaining for Pax7 and MyoD in muscles at PID7 showed that Prmt7-deficient muscles had more muscle progenitors expressing only Pax7 (Pax7^+^/MyoD^−^) while had less differentiating Pax7^−^/MyoD^+^ population, compared to wildtype (Fig. 2h, i). These data further support that Prmt7 deficiency causes impaired myogenic differentiation contributing to delayed muscle regeneration.

### Prmt7 induces MyoD-mediated myogenic differentiation and transcription

**Fig. 3:**
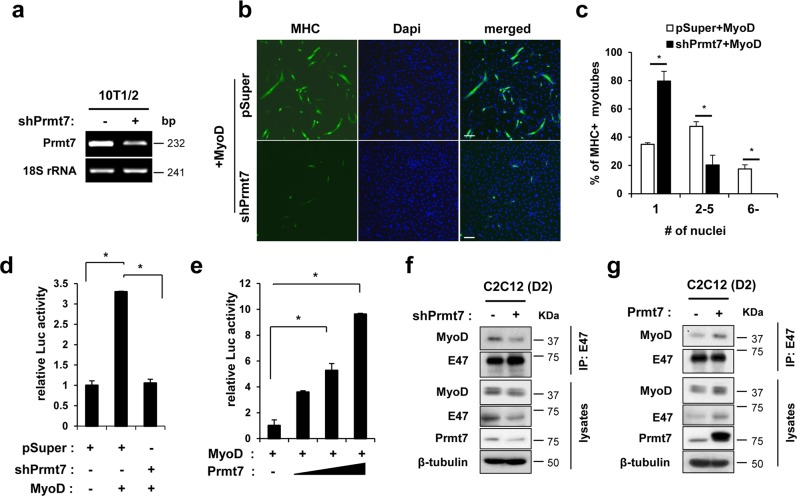
Prmt7 Facilitates MyoD Activation through Its Heterodimerization with E47. **a** Semi-PCR analysis for Prmt7 in control and Prmt7-depleted 10T1/2 mouse embryonic fibroblasts. **b** Control pSuper or shPrmt7 expressing 10T1/2 cells were transfected with MyoD and induced to differentiate for 3 days followed by immunostaining for MHC. Representative images are shown. Size bar, 100μm. **c** The quantification of MHC-positive cells with indicated nuclei are plotted. Values are determinants of random 10 fields from triplicates ± SD. *P < 0.05. **d** Luciferase assay with 10T1/2 cells cotransfected with MyoD-reporter, MyoD expression vectors and control or Prmt7 shRNA expression vectors. Values are means of triplicate determinants ± SD. *P < 0.05. **e** Luciferase assay with 10T1/2 cells transfected with MyoD-reporter and MyoD expression vectors with increasing amounts of Prmt7 expression vectors. Values are means of triplicate determinants ± SD. *P < 0.05. **f**, **g** Coimmunoprecipitation of pSuper and pSuper/shRNA Prmt7 **f** or pcDNA3.1-HA and Prmt7-HA **g** transfected C2C12 cells at D1 with anti-E47 antibodies and immunoblot analysis with indicated antibodies. This experiment was repeated at least 3 times with similar results

The facts that Myogenin induction is under the control of MyoD and p38MAPK activation, which is critical for the transition from proliferative state to differentiation of myoblasts led us to examine whether Prmt7 is involved in regulation of MyoD activities. Control shRNA or shPrmt7 expressing 10T1/2 mouse embryonic fibroblasts were transfected with a MyoD expression vector and induced to differentiate for 3 days, followed by MHC immunostaining (Fig. 3a, b). Prmt7-depleted cells exhibited decreased myogenic conversion induced by MyoD and formed fewer multinucleated myotubes, compared to control (Fig. 3b, c). To further assess the effect of Prmt7 on MyoD activities, 10T1/2 cells were cotransfected with MyoD-responsive E-box luciferase (E-box-luc) in combination with pSuper, pSuper plus MyoD, or shPrmt7 plus MyoD and induced to differentiate for 48 h. The expression of MyoD elevated the reporter activities about 3.5-fold in control cells, however, Prmt7-depletion abrogated the enhancement of MyoD-mediated reporter activity to basal levels (Fig. 3d). Conversely, 10T1/2 cells were cotransfected with vectors for E-box-luc, MyoD, and the increasing amount of Prmt7 (Fig. 3e). The MyoD-reporter activities were elevated with increasing Prmt7 levels. MyoD heterodimerization with E12/E47 is critical for MyoD activation [7–10]. Thus, we have examined MyoD heterodimerization with E47 in Prmt7-depleted or overexpressing C2C12 myoblasts at D1 (Fig. 3f, g). Prmt7 depletion reduced MyoD levels in E47 immunoprecipitation, while Prmt7 overexpression elevated it. These data suggest that Prmt7 is critical for MyoD activation to augment myogenic differentiation.

### Prmt7 recruits MyoD-mediated transcription factors to the *Myogenin* promoter region

**Fig. 4:**
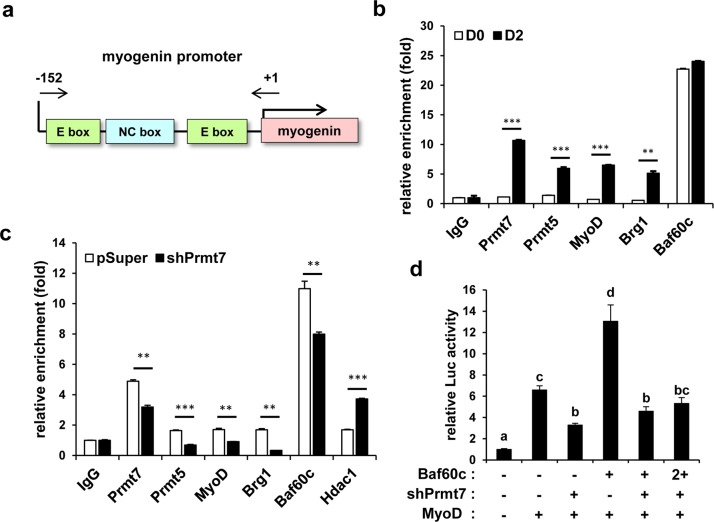
Prmt7 Upregulates Recruitment of MyoD-Mediated Transcription Factors to the Myogenin Promoter. **a** A schematic diagram of the proximal region around E-boxes from + 1 to −152 regions of the Myogenin promoter. **b** Chromatin-immunoprecipitation (ChIP) assay of the Myogenin promoter region around E-boxes in C2C12 cells at D0 or D2. Values are means of triplicate determinants ± SD. **P < 0.01, ***P < 0.001. **c** ChIP assay of the Myogenin promoter region around E-boxes in Prmt7-depleted C2C12 cells at D2. Values are means of triplicate determinants ± SD. **P < 0.01, ***P < 0.001. **d** Luciferase assay with 10T1/2 cells cotransfected with MyoD-reporter, MyoD expression vectors and Prmt7 shRNA or Baf60c expression vectors. Values are means of triplicate determinants (ANOVA Tukey, *P* *<* 0.05)

MyoD-mediated transcription requires the recruitment of cofactors such as histone modifying enzymes and SWI/SNF chromatin remodeling complexes [20]. Next, we determined whether Prmt7 is recruited to the MyoD-responsive E-box sequences in the *Myogenin* promoter (the region between −152 and +1) by chromatin immunoprecipitation (ChIP) assays (Fig. 4a). Together with MyoD, Prmt5 and Brg1, Prmt7 was greatly enriched in C2C12 cells at D2, compared to C2C12 cells at D0, while Baf60c was recruited to this region at both time points (Fig. 4b). Prmt7-depleted cells exhibited significantly blunted enrichment of MyoD, Brg1, Baf60c, and Prmt5, while more HDAC1 was recruited to the *Myogenin* promoter region, likely contributing to the inhibition of Myogenin expression (Fig. 4c). Since Baf60c is critical for MyoD-mediated transcription [14], 10T1/2 cells were cotransfected with the E-box-luc in combination with MyoD, Baf60c or shPrmt7 and induced to differentiate for 24 h. Baf60c increased MyoD-reporter activities in pSuper-transfected cells, while Prmt7 depletion abrogated this increase by Baf60c (Fig. 4d). These data suggest that Prmt7 regulates myoblast differentiation through promoting MyoD transcription activities.

### Prmt7 interacts with and methylates p38MAPKα which is required for MyoD activation

**Fig. 5:**
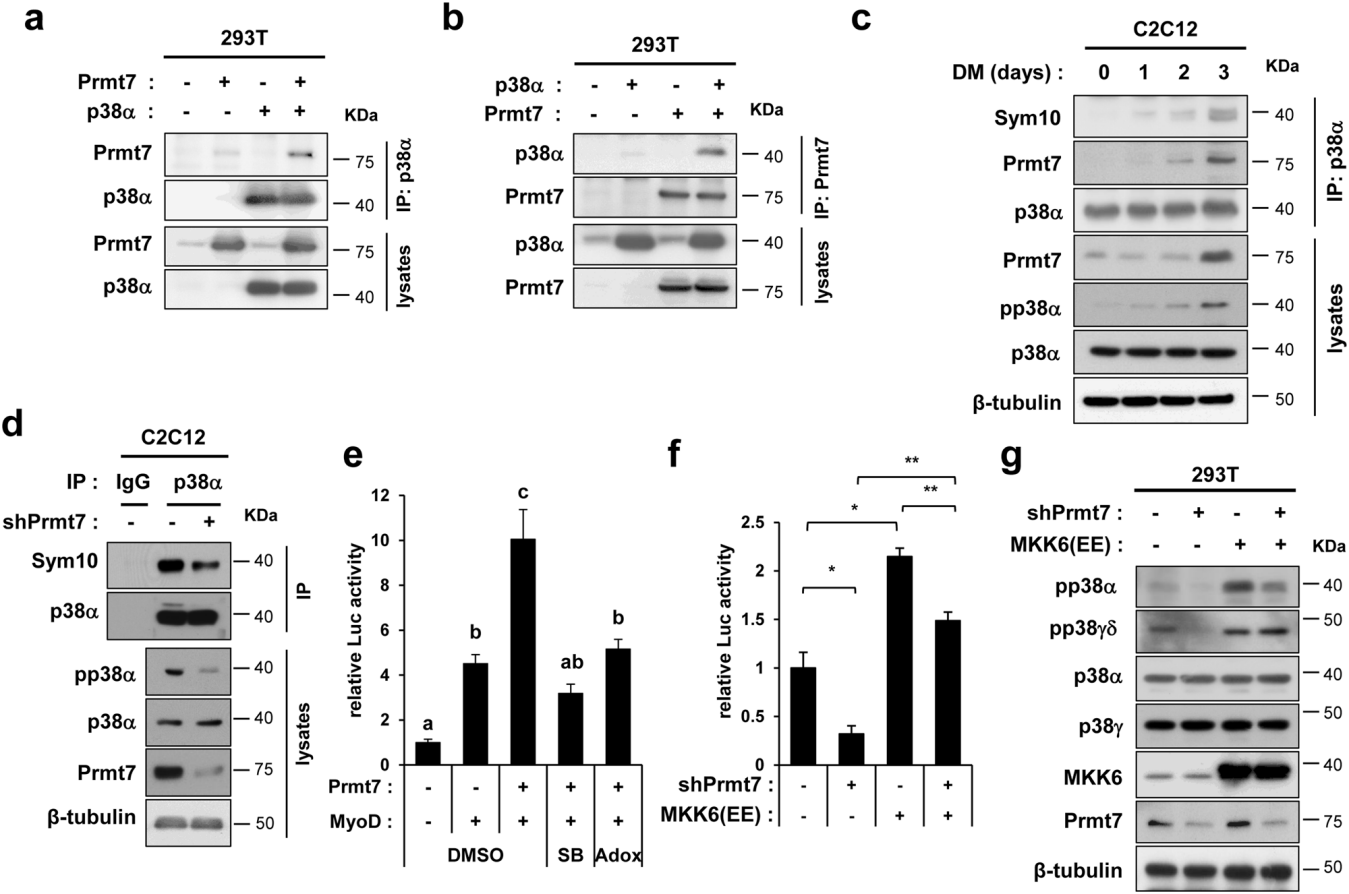
Prmt7 Interacts with and Methylates p38α. **a**, **b** Lysates of 293 T cells transfected with control, p38α and/or Prmt7 vectors as indicated were subjected to immunoprecipitation with anti-p38α or Prmt7 antibodies and immunoblotting. **c** Coimmunoprecipitation with anti-p38α antibodies in C2C12 lysates from differentiation time course. Anti-Sym10 antibody recognizing symmetrically methylated arginine residues was used to assess arginine methylation of p38α. **d** C2C12/pSuper and C2C12/shPrmt7 cells were induced to differentiate for 2 days. Anti-IgG and p38α immunoprecipitates were analyzed by immunoblotting analysis with indicated antibodies. **e** 10T1/2 cells were cotransfected with MyoD-reporter, MyoD and Prmt7 expression vectors and 24 h later, control (DMSO), 5 μM SB203580 or 50 μM Adox were treated for 24 h, followed by luciferase assay (ANOVA Tukey, *P* < 0.05). **f** The MyoD-reporter assay with 10T1/2 cells cotransfected with pSuper or Prmt7 shRNA and control or MKK6(EE). Values are means of triplicate determinants ± SD. *P < 0.05, **P < 0.01. **g** Lysates of 293 T cells transfected with pSuper, Prmt7 shRNA, control pcDNA or MKK6(EE) were immunoblotted with indicated antibodies

MyoD/E protein heterodimerization is induced through E protein phosphorylation by p38MAPK (p38) [13]. As previously shown, Prmt7 deficiency causes reduced p38 and ATF2 activation, leading to decreased PGC1α expression [28]. Thus, we have hypothesized that Prmt7-dependent MyoD activation is mediated by p38. Prmt7 interacts with p38α when coexpressed in 293 T cells (Fig. 5a, b). To examine p38α methylation during differentiation, C2C12 lysates were immunoprecipitated with anti-p38α antibodies, followed by immunoblotting with anti-Sym10 antibodies recognizing symmetric dimethylarginines (Fig. 5c). Sym10-p38α levels were increased during differentiation, correlating well with the concomitant increase of Prmt7 and the active-phosphorylated-p38α (pp38α) levels. In contrast, Prmt7-depleted C2C12 cells at D2 had decreased Sym10-p38α with concomitant reduction in pp38α (Fig. 5d). Furthermore, MyoD-reporter activities enhanced by Prmt7 was abrogated by the treatment with a p38 inhibitor SB203580 or a Prmts inhibitor adenosine dialdehyde (Adox), respectively (Fig. 5e). We then examined the rescue effect of MKK6(EE), an active form of an upstream kinase for p38 on E-box-luc in Prmt7 depletion (Fig. 5f). The expression of MKK6(EE) enhanced MyoD-reporter activities in both control and Prmt7-depleted cells. However, MyoD-reporter activities were still not fully restored in Prmt7-depleted cells. Consistently, MKK6(EE) robustly elevated pp38α levels in control cells, but Prmt7 depletion led to decreased pp38α levels (Fig. 5g). The level of pp38γδ was only affected in control Prmt7-depleted cells while its phosphorylation by MKK6(EE) was normal. To validate anti-pp38 antibodies, p38γ or p38α was depleted by siRNAs in C2C12 myoblasts and cells at D1 were subjected to immunoblotting. Depletion of p38γ or p38α decreased pp38γδ or pp38α levels, respectively, supporting for the antibody specificity. Interestingly, pp38α or pp38γδ levels increased in p38γ- or p38α-depleted cells, respectively (Figure S4). These results suggest a potential crosstalk between p38 isoforms. Moreover, Prmt7 was coimmunoprecipitated with p38α, but not with p38γ (Figure S5). These data suggest that Prmt7 enhances MyoD activity through p38α.

### Prmt7 activates p38α through methylation on the arginine residue 70 leading to MyoD activation

**Fig. 6:**
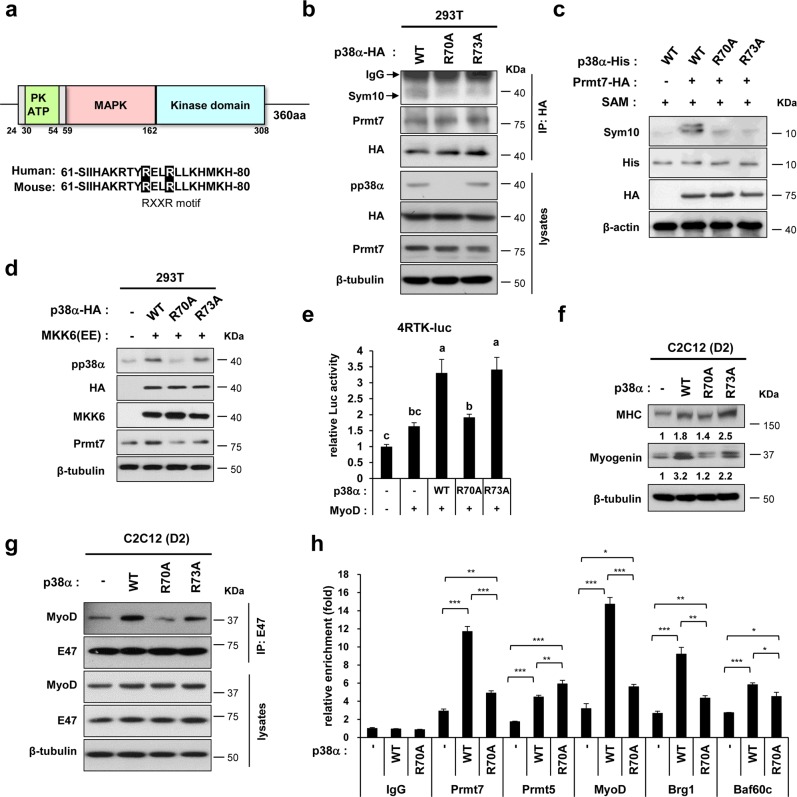
Prmt7 Methylates p38α on Arginine Residue 70 which Is Required for MyoD-Dependent Myoblast Differentiation. **a** A schematic representation of p38α protein domain. Alignment of mouse and human p38α sequences containing conserved Arg (R70)-XX-Arg (R73) residues are shaded in black. **b** Immunoprecipitation of HA-tagged wildtype (WT), R70A or R73A p38 proteins expressed in 293 T cells by using anti-HA antibodies. The methylation of p38 proteins were assessed by immunoblotting with anti-Sym10 antibodies. **c** *In vitro* methylation analysis of bacterially purified His-tagged p38α WT, R70A or R73A proteins in the presence of SAM and purified HA-Prmt7 by immunoprecipitation. The methylation status was detected by immunoblotting with anti-Sym10 antibody. **d** Lysates of 293 T cells transfected with MKK6(EE) or control, WT p38α, R70A or R73A were immunoblotted with indicated antibodies. **e** 10T1/2 cells were cotransfected with a MyoD-responsive luciferase reporter and the expression vectors for MyoD and control, WT, R70A or R73A p38α proteins. Values are means of triplicate determinants (ANOVA Tukey, *P* < 0.05). **f** Quantitative RT-PCR analysis for Myogenin levels in C2C12 cells transiently expressing control, WT, R70A or R73A p38α proteins at D2. **g** C2C12 cells transfected with WT, R70A or R73A p38α were induced to differentiate for 2 days. Anti-E47 immunoprecipitates were analyzed by immunoblotting analysis via indicated antibodies. **h** ChIP assay of the Myogenin promoter region around E-boxes in C2C12 cells transfected with WT, R70A or R73A p38α. Values are means of triplicate determinants ± SD. *P < 0.05, **P < 0.01, ***P < 0.001

p38α contains a potential consensus sequence, RXXR at arginine 70 and 73. Thus, the arginine-to-alanine mutation in RXXR at arginine 70 (R70A) and 73 (R73A) position in p38α was tested (Fig. 6a). Although both mutants showed reduced Sym10-immunoreactivity, R70A/p38α had diminished pp38α levels, while R73A/p38α had similar pp38α levels, relative to wildtype/p38α (Fig. 6b). To verify, in vitro methylation assay was performed with bacterially-expressed His-tagged p38α fragments containing amino acid (aa) 59 to aa162 as substrate with immunopurified-Prmt7-HA in presence of S-adenosyl methionine (SAM), as the methyl group donor. The methylated-p38α fragments were detected by anti-Sym10 antibody. Wildtype/p38α fragment was methylated by Prmt7 while R70A/p38α and R73A/p38α exhibited blunted methylation (Fig. 6c). Considering the facts that Prmt7 depletion reduced the effect of MKK6(EE) on p38α phosphorylation, we examined the effect of MKK6(EE) on p38α mutants. MKK6(EE) robustly elevated the level of phosphorylated-wildtype/p38α and R73A/p38α, relative to control (Fig. 6d). However, MKK6(EE) failed to increase phosphorylated R70A/p38α. Consistently, the expression of wildtype/p38α or R73A/p38α elevated MyoD-reporter activities, while R70A/p38α failed (Fig. 6e). Furthermore, wildtype/p38α and R73A/p38α enhanced Myogenin transcripts and proteins in C2C12 myoblasts, while this increase was abrogated in R70A/p38α-expressing cells (Fig. 6f, g). Consistently, the expression of wildtype/p38α or R73A/p38α increased relative MyoD levels in E47 immunoprecipitates, while R70A/p38α reduced the E47-bound MyoD levels (Fig. 6g). Finally, we have examined the effect of wildtype/p38α and R70A/p38α on the recruitment of transcription regulators to the *Myogenin* promoter region (Fig. 6h). Unlike wildtype/p38α, R70A/p38α significantly attenuated the recruitment of Prmt7, MyoD, Brg1, and Baf60c. These data suggest that Prmt7-mediated methylation of p38α at R70 is critical for p38α activation and MyoD-mediated myoblast differentiation.

## Discussion

In this study, we have investigated the regulatory mechanism of Prmt7 in muscle differentiation. Prmt7 depletion in myoblasts causes impaired myoblast differentiation, resulting from the diminishing methylation of p38α on R70. Prmt7-depleted myoblasts displayed perturbed cell cycle withdrawal upon differentiation induction, likely contributing to impaired myoblast differentiation. These data are partially consistent with the previous report showing that Prmt7 depletion impairs satellite cell differentiation and muscle regeneration [27]. This is somewhat different than the previous study reporting that Prmt7 deletion causes the cellular senescence of activated muscle stem cells which results in defective proliferation and delay in differentiation. This discrepancy is likely due to the different ages of mice used in two studies. Blanc et al. has reported that the senescence-associated regeneration defects are observed in 8-month-old adult mice while 2-month-old mice did not display same defects. Furthermore, young Prmt7-deficient mice showed impaired muscle differentiation that is similar what has been observed in our study with 4-month-old mice. The facts that Prmt7-deficient muscles had elevated levels of Pax7-expressing progenitors during regeneration and Prmt7-deficient myoblasts display delayed cell cycle withdrawal suggest that Prmt7 likely is critical for the initiation of myogenic differentiation.

The arginine dimethylation of histone subunits has been associated with modulation of gene expression in diverse biological processes [15, 16]. Especially, the dimethylation of histone H4R3 appears to be important for Prmt7-mediated gene regulation in various cellular events. Previously, Prmt7 has been shown to interact with Brg1 and SWI/SNF complex components such as Baf60 and methylates histone substrates, including histone H4R3 at the target DNA repair genes, in the DNA damage responses [1]. Furthermore, Prmt7 is implicated in neural differentiation through methylation of H4R3 and inhibition of a lysine methyltransferase MLL4 recruitment [22]. In the germinal center formation, Prmt7 influences H4R3 methylation at promoter region of Bcl6 [29]. However, nonhistone substrates for Prmt7 are not well defined [30]. In this study, we demonstrate that Prmt7 methylates and activates p38α, thereby enhancing MyoD transcriptional activation. The interaction of Prmt7 and p38α increases upon differentiation induction correlating with the gradual increase in pp38α levels. Prmt7 increases greatly at D3 when pp38α levels are robustly elevated. Although Prmt7 levels in early differentiation days are not elevated relative to the non-differentiating condition (D0), the interaction between Prmt7 and p38α increases gradually, suggesting an inductive signal for the interaction of these proteins upon differentiation. Currently the identity of this inductive signal is unclear.

As previously suggested, Prmt5 is enriched to the E-box region of the *Myogenin* promoter [19]. Therefore, it is conceivable that Prmt7 together with Prmt5 might control MyoD-transcription activities. Considering unaltered Prmt5 levels in Prmt7-deficient myoblasts, Prmt7 might be facilitating the initial recruitment of Prmt5, which might be contributing to histone dimethylation in MyoD-target genes. Prmt5 is recently shown to play a critical role in expansion of activated satellite cells which is critical for muscle regeneration [25]. Thus, it suggests that Prmt7 and Prmt5 regulate different sets of genes and differ in their ability to control cell growth and proliferation. Since Prmt5 expression decreases but persists during myoblast differentiation, Prmt7 might regulate the target specificity of Prmt5 to induce the transition of proliferative to differentiation state by activating MyoD-triggered gene expression. Further study is required for determining the functional significance of this interaction between these enzymes.

p38 signaling regulates myogenesis at several processes such as cell cycle control, MyoD dimerization with E proteins, Mef2 transcriptional activity and chromatin remodeling at muscle-specific genes including Baf60c [11–14]. Especially, p38 positively regulates MyoD activities through E protein phosphorylation, resulting in enhancement of MyoD/E protein heterodimerization [13, 31]. Previously, the opposing roles of p38α and p38γ have been proposed in MyoD-mediated myoblast differentiation [32]. p38α enhances the recruitment of MyoD and coactivators including Baf60 and Brg1 to the *Myogenin* promoter, resulting in MyoD-mediated gene expression [12, 33]. In contrast, p38γ suppresses MyoD transcriptional activity through phosphorylation, leading to enhanced recruitment of MyoD and KMT1A methyltransferase to the *Myogenin* promoter and extensive methylation of histone H3K9 [32]. A recent study showed that p38α activation interferes the interaction of MyoD and KMT1A thereby activating MyoD-mediated gene expression [34]. Based on that Prmt7 interacts specifically with p38α but not p38γ, it is tempting to speculate that p38α methylation by Prmt7 might be interfering KMT1A interaction contributing to myogenic differentiation. In addition to the direct regulation of Prmt7 on p38α activity by methylation, the data showing that Prmt7 depletion also reduces pp38γδ at the basal state suggests an indirect effect of Prmt7 in the activation of other p38 isoforms. In conclusion, we propose that Prmt7 regulates p38α activity through methylation on R70 which subsequently increases MyoD-dependent myoblast differentiation. Based on a previous [34] and our current study, it is concluded that Prmt7 plays a key role in activation and subsequent differentiation of muscle stem cells to facilitate efficient muscle regeneration.

## Experimental procedures

### Animal studies

Male and female Prmt7^<tm1a(EUCOMM)Wtsi>^ mice were purchased from Sanger Institute. All animal experiments were approved by the Institutional Animal Care and Research Advisory Committee at Sungkyunkwan University School of Medicine Laboratory Animal Research Center. Mice were backcrossed onto C57BL/6J background for at least 6 generations and maintained in C57BL6/J background and littermate wildtype controls were used for comparison with *Prmt7*^*−/−*^ mice in all experiments. To examine the skeletal muscle regeneration, mice were anesthetized with a 1–2% Isoflurane followed by cardiotoxin (CTX, 20 μM, Sigma-Aldrich, St. Louis, MO) injection into TA muscles.

### Immunostaining analysis

Freshly dissected TA muscles were snap-frozen in optimal temperature cutting (OCT) and sectioned with 7μm thickness on a cryostat microtome (Leica, Wetzlar, Germany). For immunostaining, muscle sections were fixed, permeabilized and processed for incubation with primary antibodies against Pax7 (Developmental Studies Hybridoma Bank (DSHB), Iowa, IA) or MyoD (Santa Cruz Biotechnology, Santa Cruz, CA), and secondary antibodies (Thermo fisher, Waltham, MA). Images were captured under Nikon ECLIPS TE-2000U and NIS-Elements F software (Nikon, Tokyo, Japan).

### Single myofiber isolation and culture

Extensor digitorum longus (EDL) muscles were digested with 0.2% Collagenase II (Worthington Biochemical, Lakewood, NJ) in Dulbecco modified Eagle’s medium (DMEM) medium for 60 min. Single myofibers were obtained by pipetting using a pasture pipette and then plated on Matrigel-coated 24-well plate in DMEM containing 10% horse serum and 1% penicillin/streptomycin for 12 h. Lentivirus particles were mixed in a medium in the presence of 8 μg/ml polybrene (Sigma-Aldrich, St. Louis, MO) and then added to the myofibers. Growth medium with 20% FBS and basic fibroblast growth factor (bFGF, 5 ng/ml, Invitrogen, Carlsbad, CA) was changed at 16 h post-transduction. Lentiviruses harbouring shRNA Prmt7 (shPrmt7) were generated with a modified lentiviral vector derived from pLKO.1 (Sigma-Aldrich, St. Louis, MO) in HEK293T cells using the helper plasmids pCMV-VSVG and pCMV delta 8.2 and used for infection of muscle stem cells on single myofiber.

### Cell culture, MHC immunostaining, DNA constructs and luciferase assay

Primary myoblasts, C2C12 myoblasts, 293T and 10T1/2 cells were cultured as previously described [35–37]. To induce differentiation of C2C12 myoblasts, cells at near confluence were changed from DMEM containing 15% fetal bovine serum (FBS; growth medium) to DMEM containing 2% horse serum (HS; differentiation medium) and myotube formation was observed at 2 or 3 days after differentiation. The differentiated cultures were then immunostained for anti-MHC antibodies (MF20, DSHB, Iowa, IA) and Alexa 488-conjugated secondary antibodies (Molecular Probes, Eugene, OR). The efficiency of myotube formation was quantified by counting nuclei in MHC-positive myotubes, as previously described [35, 38, 39]. Generally, at least 10 different fields were quantified and experiments were repeated at least three times with similar results. To prepare stable cell lines, C2C12 cells were transfected with pSuper or pSuper/shPrmt7 and selected with 1μg/ml puromycin (Sigma-Aldrich, St. Louis, MO) followed by pooling the colonies and analyzed. To test the specificity of anti-p38 antibodies, C2C12 cells were transfected with the control siRNA (Cat no. SN-1011, Bioneer, Seongnam, Korea), two different p38γ siRNA or three different p38α siRNA by using Lipofectamin RNAiMAX reagent (Thermo Fischer, Waltham, MA). 10T1/2 cells were culture in 10% FBS/DMEM medium and transfected with combination of pSuper or pSuper/shPrmt7 with pBabe-Puro (pBp) or pBabe-Puro/MyoD [37] using Lipofectamin 2000 (Thermo fisher, Waltham, MA). Cells were then induced to differentiate by switching into DM for 3 days, followed by immunostaining with anti-MHC antibodies.

Primary myoblasts are isolated from hindlimbs of 1 month old *Prmt7*^*+/+*^ and *Prmt7*^*−/−*^ mice and cultured, as previously described [37]. Cells were grown in F10 medium (Thermo fisher, Waltham, MA) containing 20% FBS and bFGF (2.5 ng/ml) with daily medium change. To induced differentiation, cells were plated with a high cell density and switched to DM. Luciferase assay was performed as previously described [37]. All experiments were carried out as triplicates and repeated at least three times. DNA constructs used in this study are as following: pcDNA3.1-HA, pcDNA3.1-HA-Prmt7, pSuper, pSuper/shPrmt7 [28], 4RTK-Luc, CMV-β-galactosidase, pBP, pBP-MyoD [37], pBMN-FlagBaf60c2 [40], and pcDNA3.1-MKK6(EE) [41]. The arginine to alanine mutation in p38 at the arginine residue 70 or 73 was generated by using a mutagenesis kit (Stratagene, San Diego, CA) and cloned into pcDNA3.1; pcDNA3.1-HA-p38 (WT, R70A and R73A). To generate pcDNA3.1-HA-p38γ, we have amplifed the p38γ fragment with pWZL Neo Myr Flag MAPK12 (Addgene, 20592) and subcloned into pcDNA3.1-HA.

### RNA, protein analysis and chromatin immunoprecipitation (ChIP)

Quantitative RT-PCR analysis was carried out, as described previously [42]. Total RNAs from muscle tissues and cells were extracted with total RNA extract kit (Thermo fisher, Waltham, MA). All data were normalized to the level of 18 S ribosomal RNA. The primer sequences are shown in Table 1. Western blot analysis was performed as previously described [38]. Briefly, cells were lysed in cell extraction buffer (10 mM Tris-HCl, pH 8.0, 150 mM NaCl, 1 mM EDTA, 1% Triton X-100) containing complete protease inhibitor cocktail (Roche Diagnostics, Basel, Switzerland), followed by SDS-PAGE and incubation with primary and secondary antibodies. Primary antibodies used are Prmt7, MyoD, CyclinD1, E47, p27, p38α (Santa Cruz Biotechnology, Santa Cruz, CA), Myogenin (Abcam, Cambridge, MA), MHC, Pax7 (DSHB, Iowa, IA), p38γ, Prmt4, Prmt5, (Cell Signaling Technology, Beverly, MA), pp38α, pp38γ (Thermo fisher, Waltham, MA), SYM10 (Millipore, Billerica, MA), HA (Abfrontier, Seoul, Korea) MKK6 and β-tubulin (Zymed, South San Francisco, CA). Immunoprecipitation were performed as described previously [43, 44]. Briefly, precleared cell extracts were incubated with primary antibodies overnight at 4 °C, followed by incubation with protein A or G-agarose beads (Roche Diagnostics) for 1 h and washing three times with cell extraction buffer. Precipitates were analyzed by western blotting. ChIP assay was carried out as previously described [45]. Antibodies used for ChIP included rabbit IgG (Millipore, Billerica, MA), Prmt7 (GeneTex, Irvine, CA), Prmt5 (Cell Signaling Technology, Beverly, MA), MyoD, Baf60c (Santa Cruz Biotechnology, Santa Cruz, CA), Brg1 and HDAC1 (Abcam, Cambridge, MA). The primers used to amplify the Myogenin promoter region between +152 to +1 are listed in Table 1. All primer sequences are listed Table 1.

**Table 1:** The Primers used in this study

Name	Sequence	
18S rRNA	F	AGGGGAGAGCGGGTAAGAGA
	R	GGACAGGACTAGGCGGAACA
Prmt7	F	TTCCCACAGCGGGCATTAT
	R	TGTAGCATGTCGGCATAGGA
Pax7	F	GAGTTCGATTAGCCGAGTGC
	R	CGGGTTCTGATTCCACATCT
MyoD	F	GATGGCATGATGGATTACAGCGGC
	R	GTGGAGATGCGCTCCACTATGCTG
Myogenin	F	ATCTCCGCTACAGAGGCGGG
	R	TAGGGTCAGCCGCGAGCAAA
eMHC	F	CTGGAGTTTGAGCTGGAAGG
	R	CAGCCTGCCTCTTGTAGGAC
E-box (−152 ~ +1) for ChIP	F	GAATCACATGTAATCCACTGGA
	R	ACGCCAACTGCTGGGTGCCA
shRNA mouse Prmt7	Sense	GATCCCGCATGACAAAGACAGAAATATTTTCAAGAGAAATATTTCTGTCTTTGTCATGTTTTTTGGAAA
siRNA mouse p38α #1	Sense	GACCAAGAAGAAAUGGAGU
siRNA mouse p38α #2	Sense	CUGAACAACAUCGUGAAGU
siRNA mouse p38α #3	Sense	CACGUUCAGUUUCUCAUCU
siRNA mouse p38γ ♯1	Sense	ACACCAAAUGGCUGGUUCG
siRNA mouse p38γ ♯2	Sense	ACAUAUCCUGUCAUCUCAC

### In vitro methylation assay

In vitro methylation assay was performed as previously described [46]. Recombinant His-tagged p38α fragments spanning from amino acid 59 to 162 in the wildtype or arginine to alanine mutant forms (R70A or R73A) were generated using pET21c vectors, transformed to BL21 (DE3) *E. coli* cells and induced by treating 0.5 mM IPTG (isopropyl-β-D-thiogalactopyranoside). His-tagged recombinant proteins were purified using Ni^2+^-NTA His bind Resin (Millipore, Billerica, MA) and dialyzed, followed by concentration using Amicon ultra centrifugal filter (Millipore, Billerica, MA). 1 μg of recombinant His-p38α fragment were incubated with beads bound to control or PRMT7-HA and 20μM S-(5′-adenosyl)-l-methionine chloride dihydrochloride (SAM) (Sigma-Aldrich, St. Louis, MO) in reaction buffer (20 mM Tris–HCl, pH 8.0, 200 mM NaCl, 0.4 mM EDTA) for 1 h at 37 °C. After washing the beads, reaction samples were subjected to SDS-PAGE and western blot with anti-SYM10 antibody to analyze the methylation status.

### Statistical analyses

Values are expressed as means ± SD or ± SEM, as indicated in the figure legends. Statistical significance was calculated using paired or unpaired two-tailed Student’s *t*-test. Differences were considered statistically significant at or under values of *P* *<* 0.05. For comparison between multiple groups, statistical significance was tested by ANOVA test using SPSS (12.0 version: SPSS, Chicago, IL).

## Supplementary information

Supplementary information

Figure S1

Figure S2

Figure S3

Figure S4

Figure S5
