# Supplementary material for: Prmt7 promotes myoblast differentiation via methylation of p38MAPK on arginine residue 70
Source: Cell Death Differ. 2019 Jun 26;27(2):573–86. doi: 10.1038/s41418-019-0373-y (PMC7206020; doi:10.1038/s41418-019-0373-y)
Supplement: Supplementary file 1 — Supplementary information [file 41418_2019_373_MOESM1_ESM.docx]

**Supplementary figure legends**

**Supplementary Fig. 1: The quantification of immunoblotting analysis.** (**a**) Quantification of blots from three experiments similarly performed as shown in panel **Figure 1a**. The signal intensity of Prmt7 was quantified, and the relative values were normalized to β-tubulin. The values of control sample were set to 1.0. Values represent the means of triplicate determinations ± 1 SD. ^*^*p* < 0.05, ****P* < 0.001. (**b**) Quantification of blots from three experiments similarly performed as shown in panel **Figure 1b**. The signal intensity of MyoD, E47, Myogenin and MHC was quantified, and the relative values were normalized to β-tubulin. The values of control sample were set to 1.0. Values represent the means of triplicate determinations ± 1 SD. ***P* < 0.01, ****P* < 0.001. **(c and d)** Quantification of blots from three experiments similarly performed as shown in panel **Figure 1g and 1h**, respectively. The signal intensities were quantified, and the relative values were normalized to β-tubulin. The values of control sample were set to 1.0. Values represent the means of triplicate determinations ± 1 SD. **P* < 0.05, ***P* < 0.01, ****P* < 0.001.

**Supplementary Fig. 2: Prmt7 deficiency causes impaired myoblasts differentiation.** qPCR analysis for MyoD and Myogenin in *Prmt7^+/+^* and *Prmt7^-/-^* primary myoblasts. Values are means ± SEM. ***P* < 0.01, ****P* < 0.001.

**Supplementary Fig. 3: Prmt7 deficient myoblasts display altered proliferation** (**a**) Representative images of single myofibers treated with control or Prmt7 shRNA lentivirus on single myofibers isolated from 3-month-old mice and cultured for 72 hours, followed by immunostaining for Pax7. (**b**) The qRT-PCR analysis for Prmt7 expression in control or Prmt7 shRNA expressing single myofibers as shown panel **a**. (**c**) Quantification of Pax7-positive clusters shown in panel **a**. Values are means ± SD. **P* < 0.05 (n = 10). (**d**) FACS analysis for monitoring the cell cycle profile in *Prmt7^+/+^* and *Prmt7^-/-^* primary myoblasts at D12 hours.

**Supplementary Fig. 4: Immunoblotting analysis for the specificity of pp38 antibodies.** Note that p38γ or p38α was depleted by each siRNA in C2C12 myoblasts, supporting for the specificity of the pp38 and pp38α antibodies. C2C12 cells were transfected with the control siRNA and two different p38γ siRNA (**a**) or three different p38α siRNA (**b**) at D1.

**Supplementary Fig. 5: Prmt7 interacts with p38α, but not p38 **Lysates of 293T cells transfected with vectors for control, p38α or p38γ and Prmt7 as indicated were subjected to immunoprecipitation with Prmt7 and immunoblotting.
