# Supplementary figures and images for: Prmt7 promotes myoblast differentiation via methylation of p38MAPK on arginine residue 70
Source: Cell Death Differ. 2019 Jun 26;27(2):573–86. doi: 10.1038/s41418-019-0373-y (PMC7206020; doi:10.1038/s41418-019-0373-y)

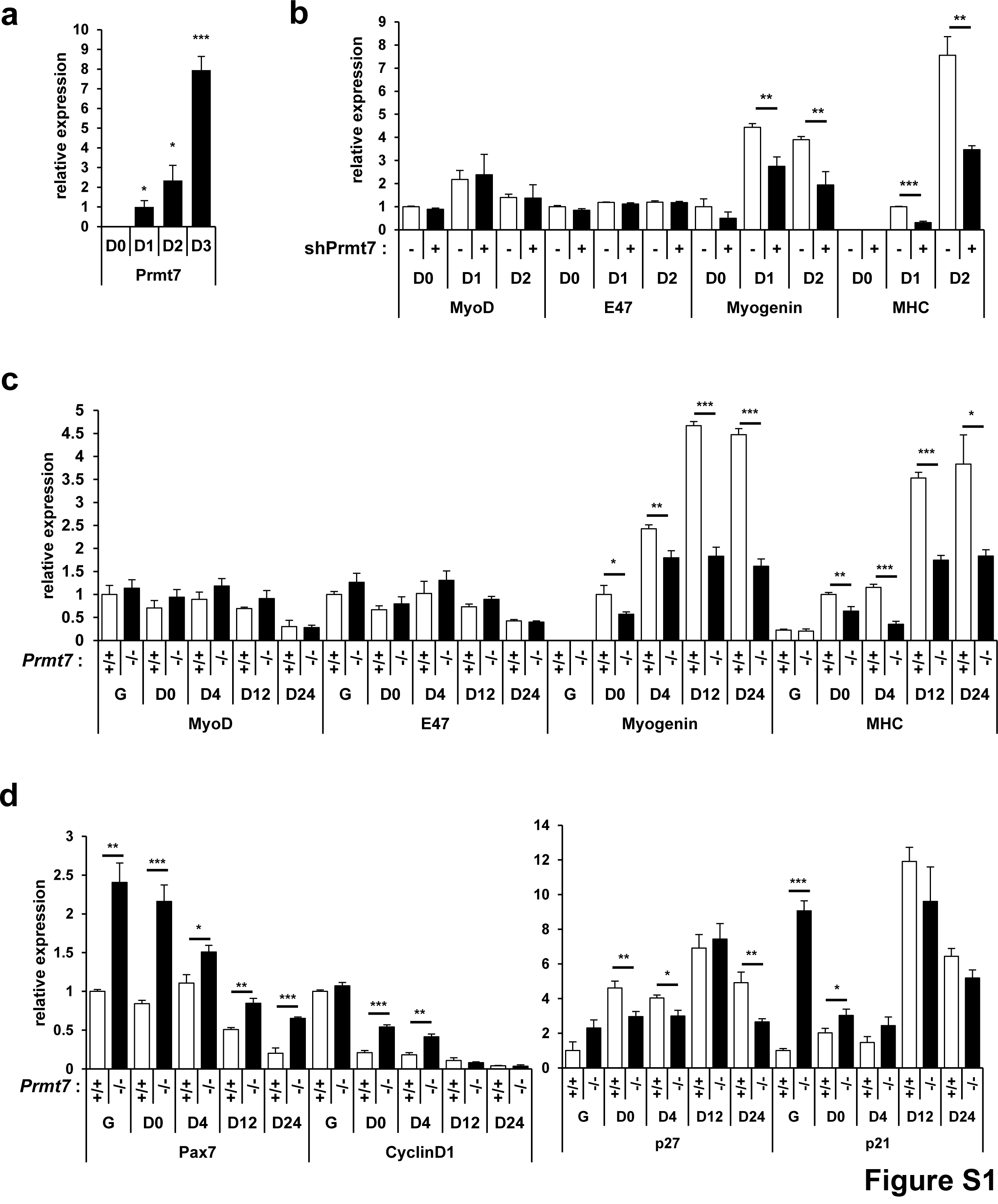

Supplement: Supplementary file 2 — Figure S1 [file 41418_2019_373_MOESM2_ESM.tif]

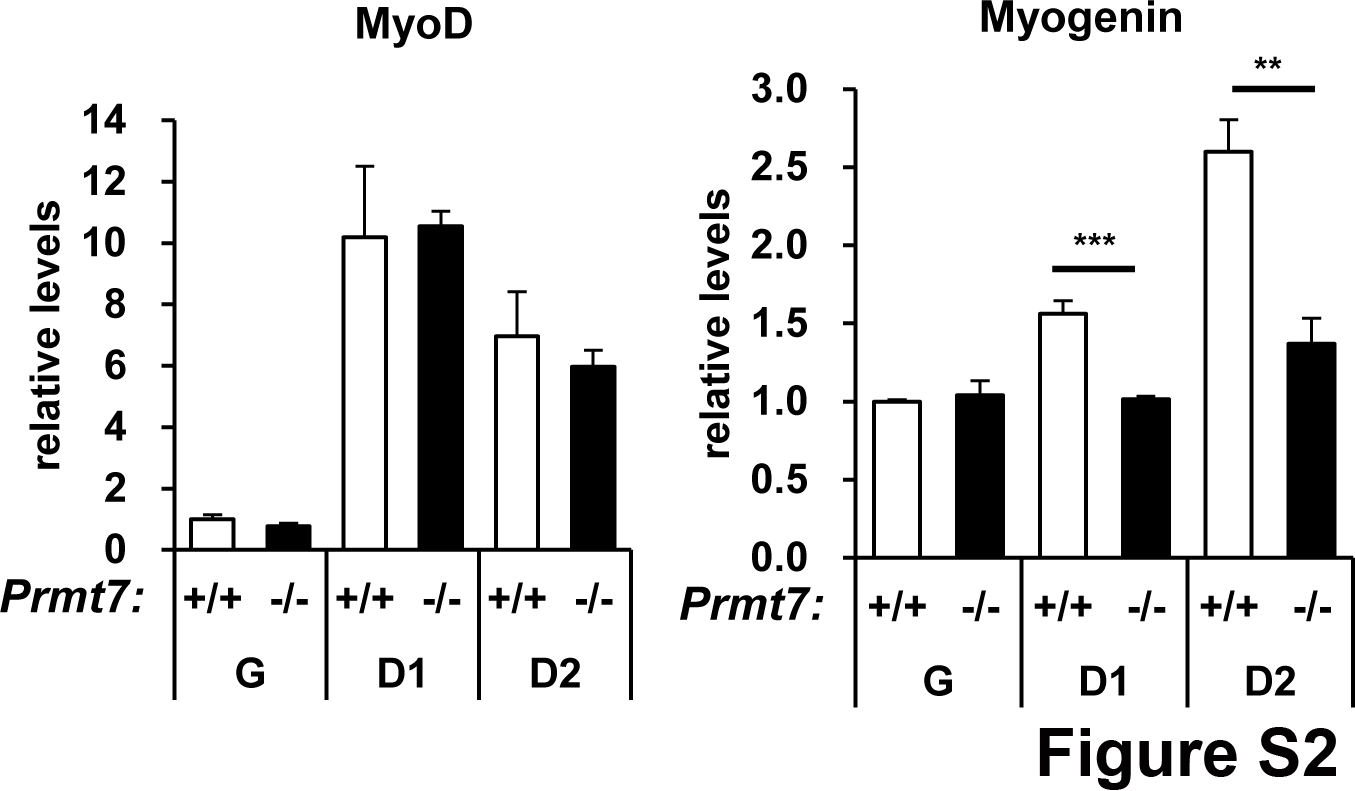

Supplement: Supplementary file 3 — Figure S2 [file 41418_2019_373_MOESM3_ESM.tif]

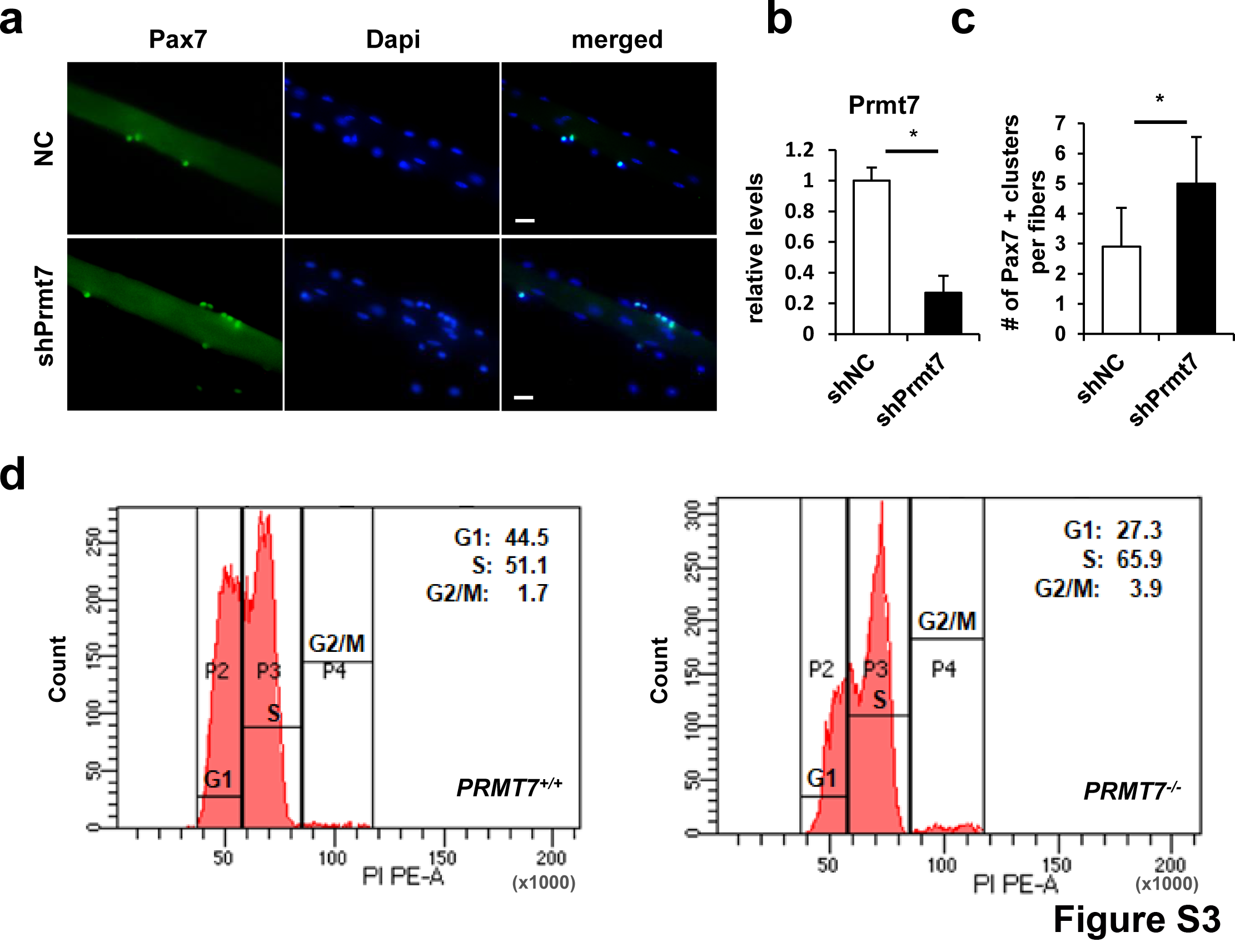

Supplement: Supplementary file 4 — Figure S3 [file 41418_2019_373_MOESM4_ESM.tif]

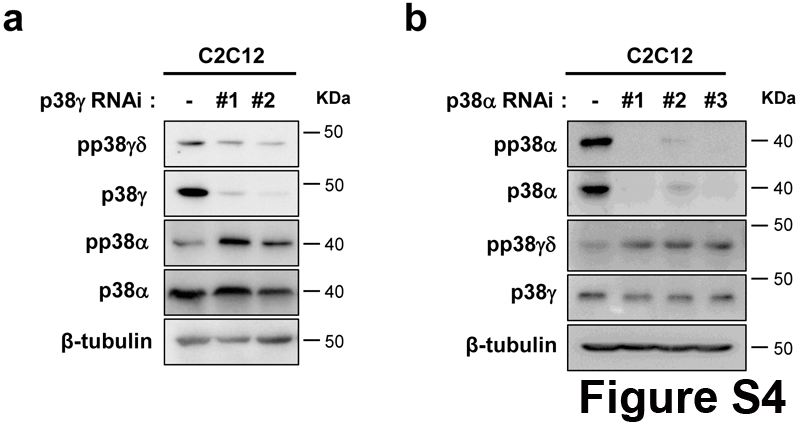

Supplement: Supplementary file 5 — Figure S4 [file 41418_2019_373_MOESM5_ESM.tif]

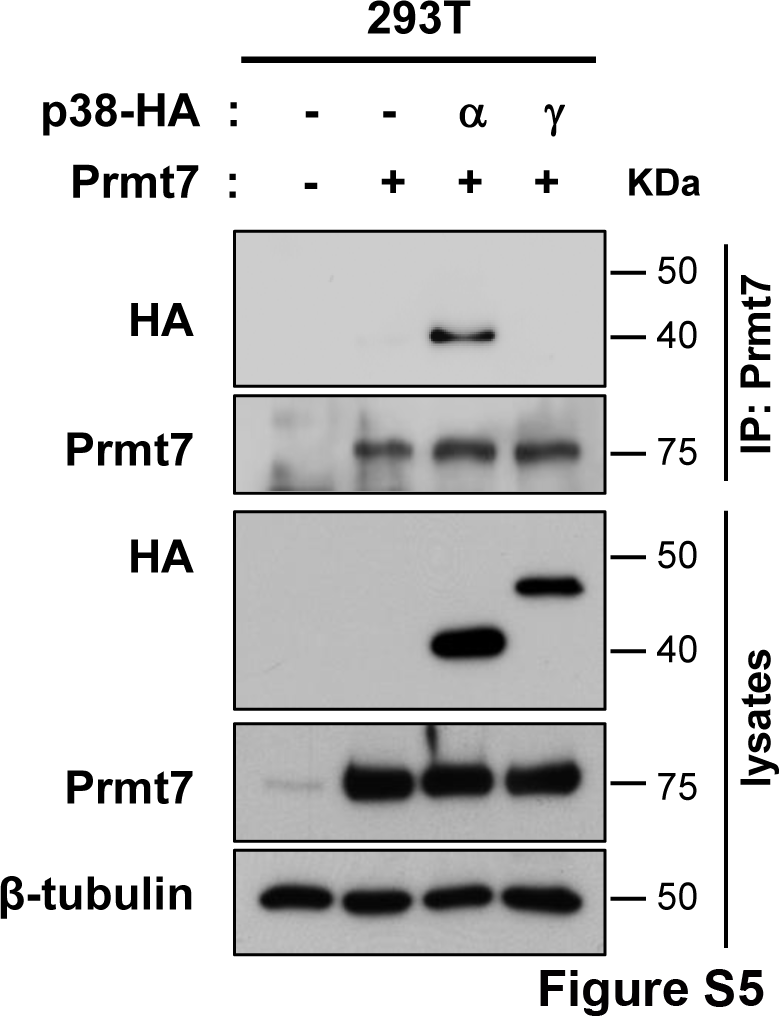

Supplement: Supplementary file 6 — Figure S5 [file 41418_2019_373_MOESM6_ESM.tif]
